# Technology for nursing care in a Maternal Intensive Care Unit: a methodological study

**DOI:** 10.1590/0034-7167-2023-0202

**Published:** 2024-05-27

**Authors:** Fabíola Nunes de Sá, Jéssica Lourenço Carneiro, Linicarla Fabiole de Souza Gomes, Joselany Afio Caetano, Ana Karina Bezerra Pinheiro, Ana Kelve de Castro Damasceno

**Affiliations:** I Universidade Federal do Ceará, Maternidade Escola Assis Chateaubriand. Fortaleza, Ceará, Brazil; II Empresa Brasileira de Serviços Hospitalares. Fortaleza, Ceará, Brazil; III Universidade Federal do Ceará. Fortaleza, Ceará, Brazil

**Keywords:** Nursing, Nursing Process, Intensive Care Units, Nursing Methodology Research, Maternal Health, Enfermería, Proceso de Enfermería, Unidades de Cuidados Intensivos, Investigación Metodológica em Enfermería, Salud Materna, Enfermagem, Processo de Enfermagem, Unidades de Terapia Intensiva, Pesquisa Metodológica em Enfermagem, Saúde Materna

## Abstract

**Objectives:**

to develop and validate a nursing care plan in a Maternal Intensive Care Unit.

**Methods:**

a methodological study, developed in stages: integrative review; Nursing History construction; care plan restructuring; appearance and content validity by judges.

**Results:**

the history was organized into sections: Identification; Basic Human Needs; Physical Examination; and Assessment of Basic Human Needs. A care plan was restructured with 34 diagnoses, organized according to basic human needs. A satisfactory level of appearance validity of the history and care plan was obtained (Concordance Index varying between 86.3 and 100 for both instruments), and content validity with average indexes of 90.8 and 92.8, respectively. Thirty-four diagnoses, their interventions and nursing actions were consolidated.

**Conclusions:**

the instruments were considered relevant and pertinent in terms of appearance and content, and their use in the institution under study as well as in other similar services may be recommended.

## RESUMO

**Objetivos:**

desenvolver e validar um plano de cuidado de enfermagem em Unidade de Terapia Intensiva Materna.

**Métodos:**

estudo metodológico, desenvolvido em etapas: revisão integrativa; construção do Histórico de Enfermagem; reestruturação do plano de cuidados; validação de aparência e conteúdo por juízes.

**Resultados:**

o histórico foi organizado em seções: Identificação; Necessidades Humanas Básicas; Exame Físico; e avaliação das Necessidades Humanas Básicas. O plano de cuidado foi reestruturado com 34 diagnósticos, organizados segundo as necessidades humanas básicas. Obteve-se nível satisfatório de validação de aparência do histórico e plano de cuidados (Índice de Concordância variando entre 86,3 e 100 para ambos os intrumentos), e validação de conteúdo com média dos índices de 90,8 e 92,8, respectivamente. Foram consolidados 34 diagnósticos, suas intervenções e ações de enfermagem.

**Conclusões:**

os instrumentos foram considerados relevantes e pertinentes quanto aparência e conteúdo, podendo ser recomendada sua utilização na instituição em estudo, bem como em outros serviços semelhantes.

## RESUMEN

**Objetivos:**

desarrollar y validar un plan de cuidados de enfermería en una Unidad de Cuidados Intensivos Materno.

**Métodos:**

estudio metodológico, desarrollado en etapas: revisión integrativa; construcción de la Historia de la Enfermería; reestructuración del plan de atención; validación de apariencia y contenido por parte de los jueces.

**Resultados:**

la historia se organizó en secciones: Identificación; Necesidades Humanas Básicas; Examen físico; y Evaluación de las Necesidades Humanas Básicas. Se reestructuró el plan de atención con 34 diagnósticos, organizados según las necesidades humanas básicas. Se obtuvo un nivel satisfactorio de validación de apariencia de la historia y plan de cuidados (Índice de Acuerdo que varía entre 86,3 y 100 para ambos instrumentos), y de contenido con índices promedio de 90,8 y 92,8, respectivamente. Se consolidaron 34 diagnósticos, sus intervenciones y acciones de enfermería.

**Conclusiones:**

los instrumentos fueron considerados relevantes y pertinentes en cuanto a apariencia y contenido, pudiéndose recomendar su uso en la institución en estudio, así como en otros servicios similares.

## INTRODUCTION

The main reasons for admission to the Intensive Care Unit (ICU) of a public maternal and child reference hospital in northeastern Brazil were hypertensive syndromes of pregnancy (62.6%), hemorrhagic syndromes (9.8%) and puerperal infection (3.3%)^(1)^. Other studies showed pre-eclampsia, hemorrhage and sepsis^(2)^. The pregnancy cycle postpartum phase was postpartum, equivalent to 75.6%^(1)^.

The three main etiologies for maternal death in the world are postpartum hemorrhage and gestational hypertension and its complications^(3)^. In Brazil, the main cause of maternal death is hypertensive complications and this appears to be the pattern throughout Latin America^(3)^. In some cases, women require ICU support, as they require special care and specific knowledge from professionals in order to meet their needs^(3)^. The nursing team requires critical and rapid assessments, comprehensive care plans, well-coordinated services with other health professionals as well as effective and convenient discharge planning^(3)^.

In this regard, care planning must be carried out using the Nursing Process (NP), which lists a set of activities that aim to professionalize care through work instruments that assist in decision-making to execute systematized, individualized, humanized and continuous care. In the context of the ICU, it directs nursing care and is considered essential for safe and quality care through effective and excellent care^(4, 5)^. Therefore, it is necessary to reflect, develop and implement a care plan that favors the incorporation of this method into practice.

In view of this, the question arises: does a nursing care plan based on basic human needs, as proposed by Horta, have validity potential for application as a tool to support the NP in a maternal ICU?

The need to perform a validated NP, aimed at obstetric patients admitted to the ICU, and the scarcity of literature regarding the proposed theme were motivators for carrying out the research.

## OBJECTIVES

To develop and validate a technology for nursing care in a Maternal ICU, based on basic human needs^(6)^.

## METHODS

### Ethical aspects

The present study was carried out in accordance with the standards of Resolution 466 of December 12, 2012 of the Brazilian National Health Council, and was approved by the Research Ethics Committee of the *Maternidade Escola Assis Chateaubriand* (MEAC), whose opinion is attached to this submission.

### Study design, period and location

This is a methodological study, carried out between January and December 2020, to develop technology for nursing care in a Maternal ICU based on the Theory of Basic Human Needs (TBHN)^(6)^, divided into four stages: 1) integrative literature review on the NP for women in the pregnancy-puerperal cycle in the maternal ICU; 2) Nursing History (NH) construction aimed at obstetric patients in the ICU; 3) adaptation of the Gomes instrument^(7)^ for use in the Maternal ICU based on integrative review results; 4) instrument appearance and content validity by experts. Data collection was in electronic format, with the research carried out at MEAC, in Fortaleza, Ceará.

### Population and sample

In this study, we chose to use the recommendation of Bolfarine and Bussab^(8)^ of 22 experts who were selected for convenience and by the network or snowball method, through the *Curriculum Lattes* and/or by recommendation from other professionals in the field.

The first contact was made by sending an invitation letter by email. Subsequently, the initial version of NP in PDF format and the appearance and content validity questionnaire were sent, which was prepared in electronic format using Google Forms®, which included the Informed Consent Form (ICF), where each evaluator, after reading the term, marked the “accepted” option to continue participating in the research. A total of 50 invitations to participate were sent, receiving acceptance from 22 experts, 12 from care and ten from teaching.

To validate appearance and content, a questionnaire organized with a Likert-type scale was used, with five degrees of appreciation: (1) Completely agree; (2) Partially agree; (3) Indifferent; (4) Partially disagree; (5) Totally disagree. For each item scored with (4) or (5), experts were asked to describe the justifications for adopting these scores so that an analysis of these justifications could also be carried out and the necessary adjustments made.

### Study protocol

Stage 1 – the stages were followed^(9)^: 1) problem identification; 2) search in literature; 3) data assessment; 4) data analysis; 5) presentation. The research question was constructed using the PICO strategy, where Population was pregnant and parturient women, Intervention was the NP and Outcomes were the nursing diagnoses and interventions in the ICU. The following question emerged: what is the evidence of application of NP in women in the pregnancy-puerperal cycle and their diagnoses and nursing interventions in obstetric ICU?

The literary search was carried out in pairs in the Cumulative Index to Nursing and Allied Health Literature (CINAHL), Medical Literature Analysis and Retrieval System online (MEDLINE), Latin American and Caribbean Literature in Health Sciences (LILACS), Scopus and Health Source databases.

After this selection, a consultation was carried out on the website in the beta version of the Health Sciences Descriptors (DeCS) and Medical Subject Headings (MeSH) for CINAHL, LILACS and Scopus and for MEDLINE and Health Source (EBSCO), in order to identify the controlled descriptors and keywords using the Boolean operators “OR” and “AND”, as described in the search strategy in Chart 1.

**Chart 1:** Search strategy with descriptors controlled by search platform. Fortaleza, Ceará, Brazil, 2020

**Databases**	**P**	**I/C**	**O**
	*Gestante, Gestação, Puérpera, Parturiente, Mulher gestante; Gestantes*	“*Processo de enfermagem*”	*Terminologia Padronizada em Enfermagem; Diagnósticos de enfermagem; intervenções de enfermagem; unidade de terapia intensiva.*
**CINAHL**	(MH “Expectant Mothers”) OR (MH “Pregnancy”) OR (MH “Postnatal Period”) OR (MH “Puerperium”)	(MH “Nursing Process”) OR (MH “Nursing Care”) OR (MH “Nursing Care Plans”) OR (MH “Nursing Care Plans, Computerized”) OR (MH “Patient Care Plans”)	(MH “Nursing Diagnosis”) OR (MH “NANDA Nursing Diagnoses”) OR (MH “Nursing Interventions”) OR (MH “Nursing Classification”) AND (MH “ Intensive Care Units”) OR (“Critical Care Nursing”)
**MEDLINE**	(MH “Pregnant Women”) OR (MH “Peripartum Period”) OR (MH “Pregnancy”) OR (MH “Postpartum Period”) OR “Puerperium”	(MH “Nursing Process”) OR (MH “Nursing Care”) OR (MH “Patient Care Planning”) OR (MH “Patient Care Management”)	(MH “Nursing Diagnosis”) OR (MH “NANDA Nursing Diagnoses”) OR (MH “Nursing Interventions”) OR (MH “Nursing Classification”) AND (MH “ Intensive Care Units”) OR (“Critical Care Nursing”)
**LILACS**	(“Pregnant Women”) OR (“Peripartum Period”) OR (“Pregnancy”) OR (“Postpartum Period”) OR “Puerperium”	(“Nursing Process”) OR (“Nursing Care”) OR (“Patient Care Planning”) OR (“Patient Care Management”)	(MH “Nursing Diagnosis”) OR (MH “NANDA Nursing Diagnoses”) OR (MH “Nursing Interventions”) OR (MH “Nursing Classification”) AND (MH “ Intensive Care Units”) OR (“Critical Care Nursing”)
**Scopus**	(“Pregnant Women”) OR (“Peripartum Period”) OR (“Pregnancy”) OR (“Postpartum Period”) OR “Puerperium”	(“Nursing Process”) OR (“Nursing Care”) OR (“Patient Care Planning”) OR (“Patient Care Management”)	(MH “Nursing Diagnosis”) OR (MH “NANDA Nursing Diagnoses”) OR (MH “Nursing Interventions”) OR (MH “Nursing Classification”) AND (MH “ Intensive Care Units”) OR (“Critical Care Nursing”)
**Health Source**	(“Pregnant Women”) OR (“Peripartum Period”) OR (“Pregnancy”) OR (“Postpartum Period”) OR “Puerperium”	(“Nursing Process”) OR (“Nursing Care”) OR (“Patient Care Planning”) OR (“Patient Care Management”)	(MH “Nursing Diagnosis”) OR (MH “NANDA Nursing Diagnoses”) OR (MH “Nursing Interventions”) OR (MH “Nursing Classification”) AND (MH “ Intensive Care Units”) OR (“Critical Care Nursing”)

*P – Population; I/C – Intervention/Comparison; O – Outcome.*

Articles published in full, available electronically, without language restrictions, published on any date, whose results provide information on nursing diagnoses and interventions, were included. Repeated articles were excluded; theses, dissertations, monographs, editorials, manuals and books.

Stage 2 – a data collection instrument regarding NH was structured, based on the instrument already used in the unit, which is common to all sectors of the hospital, in order to structure the interview, including personal data, vital signs and a complete physical examination based on the basic human needs of Wanda Horta’s proposed theoretical model, according to the 35 levels of needs that make up the psychobiological, psychosocial and psychospiritual dimensions^(6)^.

Stage 3 – the “Nursing Care Plan for High-Risk Pregnant Women” instrument was adapted, which had been developed and validated in a previous study to the reality of the Maternal ICU, previously authorized by the author^(7)^. After the conceptual definition of nursing diagnoses, diagnostic indicators were constructed (defining characteristics, related and risk factors), which were listed taking into account the profile of the target population. Subsequently, nursing interventions and actions were selected, based on the instrument being adapted, the author’s experience and research in the literature collected.

It is noteworthy that the diagnoses and interventions that were proposed for the execution of the version of the technology to be assessed for reliability, applicability and usefulness were based on the NANDA International classification system^(10)^ in the 5^th^ edition of the Nursing Outcome Classification^(11)^ (NOC) and in the 6^th^ edition of the Nursing Interventions Classification^(12)^ (NIC) as well as being structured and organized based on the 35 levels of needs of the three dimensions of TBNH^(6)^.

Stage 4 – instrument content and appearance validity was carried out with experts^(8)^.

### Analysis of results, and statistics

The Concordance Index (CI) was adopted, in which the number of times there is agreement is divided by the total number of assessments, varying between 0 and 100%. For an “adequate” or “excellent” assessment, a CI = 80% agreement was considered. CI was calculated as follows: CI= Number of “completely agree” and “partially agree” responses x 100/total number of answers. CI values between 80 and 100 were considered satisfactory, being attributed to experts’ approval regarding the permanence of assessed items.

To check whether the items on the form had an agreement greater than 80%, the Wilcoxon-Mann-Whitney test was performed with continuity correction, considering the significance level p > 0.05 and agreement proportion of 0.80 to estimate the statistical reliability of CI^(13)^.

For data analysis, the Statistical Package for the Social Sciences (SPSS) version 24.0 (SPSS Inc., Chicago, IL, USA) was used. Absolute and relative frequencies were calculated for qualitative variables as well as mean, standard deviation, quartiles, minimum and maximum for quantitative variables^(14, 15)^. Cronbach’s alpha was also calculated, which, on a scale from 0 to 1, assessed the reliability of an instrument, i.e., internal consistency, with 0.7 being the minimum acceptable value to consider this tool reliable, a value considered a reference for analysis of this research^(14, 15)^.

## RESULTS

The NH instrument was structured and presented through four data categories: I. Identification; II. Basic Human Needs; III. Physical Examination; and IV. Assessment of Basic Human Needs, which was subdivided into: 1) Affected needs; and 2) Degree of dependence on Nursing. This is the basis for implementing the care plan, as it constitutes a summary of data collection, which will guide the selection of nursing diagnoses and the prescription of care by nurses.

Regarding the Nursing Care Plan for High-Risk Pregnant Women^(7)^, of the 21 diagnoses, ten were excluded, such as obesity/overweight, nausea, impaired sleep pattern, fatigue, bathing self-care deficit, toileting self-care deficit in self-care, readiness for enhanced self-care, ineffective health maintenance, poor knowledge, anxiety.

A total of 11 diagnoses were maintained, such as acute pain, excessive fluid volume, deficient fluid volume, impaired comfort, constipation, impaired urinary elimination, impaired physical mobility, risk for infection, impaired skin integrity, impaired tissue integrity, fear.

A total of 23 diagnoses were added, such as ineffective breathing pattern, impaired gas exchange, impaired spontaneous ventilation, dysfunctional ventilatory weaning response, impaired swallowing, unbalanced nutrition: less than body requirements, risk for unstable blood glucose, diarrhea, disturbed sleep pattern, risk for falls, impaired oral mucous membrane integrity, risk for impaired skin integrity, ineffective peripheral tissue perfusion, risk for pressure injury, hyperthermia, hypothermia, risk for electrolyte imbalance, risk for aspiration, risk for shock, risk for bleeding, acute confusion, impaired religiosity, and spiritual distress.

At the end of this journey, a care instrument was constructed with 34 diagnoses, which were organized by basic human needs (psychobiological, psychosocial and psychospiritual) based on their levels of needs.

Of the 34 diagnoses, most of them cover psychobiological needs; of these, four related to oxygenation level (11.8%), three in nutrition (8.8%) and three in elimination (8.8%), one in sleep and rest (2.9%), two in locomotion (5.9%), five in skin-mucosal integrity (14.7%) and 11 in regulation (32.4%).

Within the psychosocial needs, there are three nursing diagnoses (8.8%), distributed into two levels of needs: safety (2/5.9%) and orientation in time and space (1/2.9%). Psychospiritual needs include two diagnoses (5.9%): within religious or theological needs, ethics or philosophy of life.

After the conceptual definition, the diagnostic indicators were constructed, which were listed taking into account the patients’ profile, as well as the researcher’s practical experience as a clinical nurse and nursing manager of this unit and, subsequently, the interventions and actions were selected of nursing.

In the end, the preparation of the first version of the “Nursing Care Plan for Women in the Pregnancy-Puerperal Cycle in Intensive Care” was completed for subsequent submission to the appearance and content validity process with experts.

### Content appearance and validity by experts

#### Experts participating in the study

The validity process by experts took place over a total period of 30 days, completely online. The sociodemographic data of the evaluators showed that the majority were female (18/81.8%), aged 30-39 years (14/63.6%) and resident in the northeast (19/86.3%). Regarding professional profile, the majority are in the range of 10 to 19 years of training (18/81.8%), and regarding qualifications, ten specialists (45.5%), followed by eight (36.4%) PhD holders, three (13.6%) masters holders and one (4.5%) post-doctoral holders. Regarding the area of activity, 15 (68.2%) are in care, 13 (59.1%) are in teaching and four (18.2%) are in management. It should be noted that 18 (81.8%) also work directly in assisting women in intensive care and 22 (100%) have some scientific production in the area.

Table 1 describes the face validity of the instruments, with the respective CI and Cronbach’s alpha.

**Table 1:** Distribution of data from the appearance validity of the Nursing History instrument and the nursing care plan, between January and December 2020, Fortaleza, Ceará, Brazil, 2023

Variables	Totally agree		Partially agree		Indifferent		Partially disagree		Totally disagree		CI
	n	%	n	%	N	%	n	%	n	%	
NH											
Is the instrument relevant?	20	90.9	2	9.1	0	0.0	0	0.0	0	0.0	100
Is the information presented clearly and objectively?	14	63.6	5	22.7	0	0.0	3	13.6	0	0.0	86.3
Is the information scientifically correct?	18	81.8	3	13.6	0	0.0	1	4.5	0	0.0	95.4
Are the font and size appropriate?	16	72.7	6	27.3	0	0.0	0	0.0	0	0.0	100
Are the spacing between letters adequate?	16	72.7	4	18.2	2	9.1	0	0.0	0	0.0	90.9
Does the instrument have a logical sequence?	16	72.7	3	13.6	0	0.0	2	9.1	1	4.5	86.3
Is the instrument easy to read and understand?	14	63.6	6	27.3	0	0.0	2	9.1	0	0.0	90.9
Total Cronbach’s alpha = 0.780	114	74.0	29	18.8	2	1.3	8	5.2	1	0.6	92.8
Nursing care plan											
Is the technology relevant?	18	81.8	4	18.2	0	0.0	0	0.0	0	0.0	100
Is the information presented clearly and objectively?	12	54.5	7	31.8	0	0.0	3	13.6	0	0.0	86.3
Is the information scientifically correct?	17	77.3	3	13.6	0	0.0	2	9.1	0	0.0	90.9
Are the font and size appropriate?	17	77.3	3	13.6	1	4.5	1	4.5	0	0.0	90.9
Are the spacing between letters adequate?	16	72.7	3	13.6	3	13.6	0	0.0	0	0.0	86.3
Does the technology present a logical sequence?	16	72.7	5	22.7	0	0.0	0	0.0	1	4.5	95.4
Is the technology easy to read and understand?	13	59.1	7	31.8	1	4.5	1	4.5	0	0.0	90.9
Total Cronbach’s alpha = 0.747;	109	70.8	32	20.8	5	3.2	7	4.5	1	0.6	91.3

*N – absolute value; % – relative value; CI – Concordance Index; NH – Nursing History.*

According to Table 1, all items were considered satisfactory, validating the appearance of these instruments positively. It is noteworthy that the Cronbach’s alpha value was 0.780 for history and 0.747 for care plan, which gives reliability to the instruments.

In Table 2, it is possible to visualize the validity process of the general aspects of the NH instrument based on the woman’s identification bar, the three levels of basic human needs, physical examination and assessment of basic human needs, with satisfactory CI values, providing a positive assessment of items according to the reference presented. Cronbach’s alpha presented was 0.824, giving confidence to the instrument.

**Table 2:** Distribution of content validity data for general aspects and general content validity of the technology of the Nursing History instrument, January and December 2020, Fortaleza, Ceará, Brazil, 2023

Variables	Totally agree		Partially agree		Indifferent		Partially disagree		Totally disagree		CI
	n	%	n	%	n	%	n	%	n	%	
Content validity of general aspects of the NH instrument											
Level of agreement: Identification	16	72.7	4	18.2	0	0.0	1	4.5	1	4.5	90.9
Level of agreement: Psychobiological Needs	11	50.0	7	31.8	1	4.5	2	9.1	1	4.5	81.8
Level of agreement: Psychosocial Needs	15	68.2	4	18.2	1	4.5	1	4.5	1	4.5	86.3
Level of agreement: Psychospiritual Needs	15	68.2	6	27.3	0	0.0	1	4.5	0	0.0	95.4
Level of agreement: Physical Examination	12	54.5	7	31.8	1	4.5	1	4.5	1	4.5	86.3
Level of agreement: Assessment of basic human needs	15	68.2	6	27.3	1	4.5	0	0.0	0	0.0	95.4
Total Cronbach’s alpha = 0.824	84	63.6	34	25.8	4	3.0	6	4.5	4	3.0	89.3
General content validity of the NH instrument technology											
Does the instrument meet the first stage of NP?	14	63.6	6	27.3	1	4.5	1	4.5	0	0.0	90.9
Is the instrument relevant to nurses’ clinical practice?	17	77.3	4	18.2	0	0.0	1	4.5	0	0.0	95.4
Does the instrument address psychobiological, psychosocial and psychospiritual needs?	15	68.2	5	22.7	0	0.0	2	9.1	0	0.0	90.9
Total: Cronbach’s alpha = 0.839	46	69.7	15	22.7	1	1.5	4	6.1	0	0.0	92.4

*CI – Concordance Index; NP – Nursing Process; NH – Nursing History.*

Table 2 also presents the general content validity process of the NH instrument, with the aim of verifying whether it meets the stages of NP, whether it is relevant to practice in focus in the study and whether it contemplates the three dimensions of basic human needs in Horta^(6)^. These factors presented a general CI of 91.2 and Cronbach’s alpha of 0.839, both satisfactory, validating the technology according to reference values.

Thus, the content validity of the nursing care plan will be described, which was carried out by diagnosis, in a summarized way, to facilitate reading and understanding, considering that there are 34 diagnoses validated individually. All CI and Cronbach’s alpha values achieved scores within the reference values, being satisfactorily validated.

Table 4 refers to the general content validity process of the nursing care plan technology with the aim of verifying whether it meets the NP stages, whether it is relevant to the practice in focus in the study and whether it contemplates the three dimensions of basic human needs in Horta^(6)^. These factors had an average CI of 91.5 and Cronbach’s alpha of 0.958, values that validate the items according to the validity methodology presented. Table 4 also refers to the summary of validity by need, according to the three dimensions of Horta’s basic human needs^(6)^, whose CI and Cronbach’s alpha values reached satisfactory levels, being validated in all aspects presented.

**Table 4:** Distribution of content validity data regarding the Nursing Process, relevance and basic human needs as well as the summary of content validity by need, according to the three dimensions of basic human needs in Horta^(6)^, January and December 2020; Fortaleza, Ceará, Brazil, 2023

Variables	Totally agree		Partially agree		Indifferent		Partially disagree		Totally disagree		CI
	n		n	%	n	%	n	%	n	%	
Content validity regarding the NP, relevance and basic human needs											
Does the instrument meet the steps of NP?	16	72.7	4	18.2	0	0.0	1	4.5	1	4.5	90.9
Is the instrument relevant to nurses’ clinical practice?	16	72.7	5	22.7	0	0.0	0	0.0	1	4.5	95.5
Does the instrument address psychobiological, psychosocial and psychospiritual needs?	17	77.3	4	18.2	0	0.0	0	0.0	1	4.5	95.5
Total Cronbach’s alpha = 0.958	49	74.2	13	19.7	0	0.0	1	1.5	3	4.5	94.0
Summary of content validity by necessity, according to the three dimensions of basic human needs in Horta^(6)^											
Psychobiological Needs^1^	2146	72.8	560	19.0	3	0.1	47	1.6	192	6.5	91.8
Psychosocial Needs^2^	251	76.1	51	15.5	0	0.0	6	1.8	22	6.7	91.5
Psychospiritual Needs^3^	164	74.5	38	17.3	0	0.0	4	1.8	14	6.4	91.8
Total Cronbach’s alpha1 = 0.999; Cronbach’s alpha2 = 0.994; Cronbach’s alpha3 = 0.989	2561	74.5	649	17.3	3	0.03	57	1.7	228	6.5	91.7

*CI – Concordance Index; NP – Nursing Process.*

After quantitative assessment, a qualitative analysis was carried out, in which experts’ suggestions for adapting the instruments were assessed, described in situations of attribution of valuation on a Likert scale of 4 (partially disagree) and 5 (completely disagree) points. Thus, at the end of this adjustment process, the history and nursing care plan were considered validated in content and appearance, constituting the final version of the technology for nursing care.

## DISCUSSION

The compiled findings were useful to compose the theoretical framework necessary for updating and developing the targeted instruments, with the aim of helping to carry out the Systematization of Nursing Care through the NP method. To be effective, nurses need to have mastery and ability to implement all the steps established by Resolution 358/2009 of the Federal Nursing Council^(16)^.

**Table 3:** Distribution of data from content validity of the nursing care plan technology, January and December 2020, Fortaleza, Ceará, Brazil, 2023

Assessed items	Concordance Index	Cronbach’s alpha
Nursing diagnoses		
Ineffective breathing pattern	90.9	0.975
Impaired gas exchange	91.8	0.973
Impaired spontaneous ventilation	91.8	0.977
Dysfunctional ventilatory weaning response	91.8	0.973
Impaired swallowing	91.8	0.975
Unbalanced nutrition: less than body requirements	91.8	0.976
Risk for unstable blood sugar	92.0	0.962
Impaired urinary elimination	90.9	0.974
Constipation	91.8	0.977
Diarrhea	91.8	0.977
Disturbed sleep pattern	91.8	0.975
Risk for falls	92.0	0.959
Impaired physical mobility	90.9	0.974
Impaired oral mucous membrane integrity	91.8	0.975
Impaired skin integrity	91.8	0.974
Risk for impaired skin integrity	92.0	0.956
Impaired tissue integrity	91.8	0.976
Risk for pressure injury	91.8	0.977
Ineffective peripheral tissue perfusion	92.0	0.956
Hyperthermia	92.0	0.959
Hypothermia	92.0	0.958
Risk for electrolyte imbalance	92.0	0.959
Excessive fluid volume	91.8	0.975
Deficient fluid volume	91.8	0.977
Acute pain	91.8	0.975
Risk for infection	92.0	0.962
Risk for aspiration	92.0	0.962
Risk for shock	92.0	0.960
Risk for bleeding	92.0	0.962
Fear/anxiety	91.8	0.975
Impaired comfort	91.8	0.977
Acute confusion	90.9	0.998
Impaired religiosity	91.8	0.975
Spiritual distress	91.8	0.974
General assessment	93.9	0.958

Another important aspect is the use of classification systems, which aim to standardize the language, providing sustainability to the profession through its own language. Investigating, diagnosing and intervening in the search for results implies bringing nurses closer to patients in subjective dimensions, interaction and trust, generating assistance that meets the individual needs of each human being.

A study identified that nursing diagnoses were significantly related to mortality and length of stay in ICUs. Thus, the importance of NP in these units is evident, since, through its application and evaluation, it is possible to estimate the complexity of patients as well as determine individual care needs based on a priority order of these actions^(17)^.

Thus, it is understood that diagnosed nursing confers value and differentials to nurses as a private technology of the profession essential to quality and individualized care. Of the diagnoses maintained in the developed instrument, the literature highlights acute pain, impaired physical mobility, risk for infection, impaired skin integrity and constipation, and their respective interventions, which were evidenced in a study that assessed the use of nursing diagnoses and interventions proposed for women in labor and high-risk pregnant women, and were, in a similar way, organized based on the NANDA International domains^(6, 18)^.

Regarding the nursing diagnosis of impaired physical mobility in ICUs, it is very common, due to the serious condition of patients in these sectors^(19)^.

It is noteworthy that the diagnoses referred to above comprise important levels of need in Horta’s psychobiological dimension, namely oxygenation, nutrition, elimination, sleep and rest, locomotion, skin-mucosal integrity, and regulation: thermal, hormonal, neurological, hydrosaline, electrolytic, immunological, cell growth, vascular^(6)^.

The new proposed diagnoses are also highlighted in the literature, such as ineffective breathing pattern, sleep pattern disturbance and unbalanced nutrition: less than body needs and risk for aspiration, highlighting interventions and actions, such as respiratory monitoring, and reporting that the difficulty in raising the upper part of the body, the depression of cough and swallowing reflexes and the increase in intragastric pressure can lead to the aspiration of secretions. The studies also propose actions aimed at risk for aspiration, and are also present for planning aimed at deficient fluid volume, which was maintained based on the instrument used for adaptation^(4, 20)^.

The nursing interventions and their psychobiological dimension actions used were highlighted by the literature in the area such as skin care, pressure control, supervision, infection control, circulatory precautions, positioning, care with probes and other devices, neurological monitoring, bathing, skin and hair care, oral health maintenance, airway suctioning, treatment of fever or hypothermia, eye care, venous access management, tube feeding, incision care^(21)^.

It is worth noting that these actions were evidenced in the planning of several nursing diagnoses, demonstrating the evidence of data included in the technology in the literature, which converges towards improvements in care in the area^(21)^.

With regard to psychosocial needs, the literature states that the needs identified for women in the pregnancy-puerperal cycle in the ICU are related to the predisposition of the psychosocial context in which they are inserted, making them also vulnerable to the physiological hormonal changes of pregnancy, which have an active role in this context^(22)^.

According to another important diagnostic concept included in the technology developed, fear was also evidenced in a study on nursing care in cesarean births as part of the coping and tolerance to stress domain, in addition to the concept of comfort, in the latter revealing the importance of psychosocial needs for women in the pregnancy-puerperal period^(23)^.

Generally, studies carried out focusing on intensive care have a predominance of psychobiological dimensions, with emphasis on oxygenation and nutrition levels and a low predominance of psychosocial and psychospiritual dimensions, following the example of literature, often not even mentioning the third level of need, which is essential to comprehensive care, which gives the instrument of this study a difference^(24)^.

Nursing diagnoses aimed at the psychospiritual dimension are essential, as they encompass spheres that are generally very affected in this target audience. However, the literature points out that these diagnoses are rarely mentioned in studies carried out in ICUs, with nursing care being more focused on controlling physical and social symptoms, which demonstrates, on the one hand, its deprioritization in this context, but, on the other, the innovation and completeness of the present study^(25)^.

### Study limitations

The limitation of this study is based on non-clinical validity of instruments, requiring further evaluation of their effectiveness in practice; however, it is worth highlighting that new studies can be carried out in order to test themselves with the respective target audience, filling this gap in knowledge.

### Contributions to nursing, health and public policies

As contributions to nursing and health, the study presents the potential for using technology in nursing care as well as in other institutions in the area, with the possibility of generating nursing indicators and integrating software for NP in the Maternal ICU. It also enables contributions to teaching and research as it provides new reflections on the use of NP.

## CONCLUSIONS

The technological instrument for nursing care for patients hospitalized in a Maternal ICU based on basic human needs, which includes an instrument for data collection and care technology, was constructed and considered for use by experts, being relevant in terms of appearance and content.

Even with the high assessment agreement, experts’ considerations were accepted according to the need and better viability of instruments, in search of improvements. Thus, the present study contemplates the framework not only theoretically in the maternal-puerperal area, but also for assistance, with the potential for using this technology in practice, contributing to the generation of nursing indicators and the future possibility of integrating a software for NP in Maternal ICU.

Therefore, the effective use of the Systematization of Nursing Care is recommended, in an exhaustive manner, by the profession, based on the ethical and legal aspects that govern it, using technologies that guide the NP method and characterize the profession, making care more consolidated in practice.
